# Effect of Protocatechuic Acid Ethyl Ester on Biomembrane Models: Multilamellar Vesicles and Monolayers

**DOI:** 10.3390/membranes12030283

**Published:** 2022-02-28

**Authors:** Cristina Torrisi, Giuseppe Antonio Malfa, Rosaria Acquaviva, Francesco Castelli, Maria Grazia Sarpietro

**Affiliations:** Department of Drug and Health Sciences, University of Catania, Viale Andrea Doria 6, 95125 Catania, Italy; torrisi.cristina@hotmail.it (C.T.); g.malfa@unict.it (G.A.M.); racquavi@unict.it (R.A.); fcastelli@unict.it (F.C.)

**Keywords:** protocatechuic acid ethyl ester, biomembrane model, multilamellar vesicle, monolayer, differential scanning calorimetry, Langmuir–Blodgett

## Abstract

The interactions of drugs with cell membranes are of primary importance for several processes involved in drugs activity. However, these interactions are very difficult to study due to the complexity of biological membranes. Lipid model membranes have been developed and used to gain insight into drug–membrane interactions. In this study, the interaction of protocatechuic acid ethyl ester, showing radical-scavenging activity, antimicrobial, antitumor and anti-inflammatory effects, with model membranes constituted by multilamellar vesicles and monolayers made of DMPC and DSPC, has been studied. Differential scanning calorimetry and Langmuir–Blodgett techniques have been used. Protocatechuic acid ethyl ester interacted both with MLV and monolayers. However, a stronger interaction of the drug with DMPC-based model membranes has been obtained. The finding of this study could help to understand the protocatechuic acid ethyl ester action mechanism.

## 1. Introduction

Studies on the interactions of cell membranes with drugs are of interest to understanding the action of molecular mechanisms. However, the processes involved in the interaction between a drug and biomembranes are very complex, and biomembranes are very complicated structures; as a consequence, interactions between drug and biomembrane are difficult to study and understand. For this reason, biomimetic model cell membranes, such as liposomes and phospholipid monolayers, are widely used to study such interactions [1,2].

Phenolic acid compounds have attracted attention because of their beneficial effects on human health. They are widespread in edible plants, vegetables, and fruits that show a lot of biological and pharmacological activities, such as antioxidant, anti-inflammatory, antibacterial, and antiproliferative effects on several types of cancer cells in vitro [3,4,5,6,7]. In comparison with other plant phenolics, free phenolic acids are relatively water-soluble; however, their low bioavailability limits their practical application as health-promoting substances in humans. Free phenolic acids are quickly metabolized and eliminated in the urine after ingestion, which further limits their bioavailability. Therefore, chemical modification of phenolic acids, especially increasing their lipophilicity, could help in achieving the condition required for their biological activity and improving their action [8]. This case could involve the esterification of PCA in protocatechuic acid ethyl ester (PCAEE). The introduction of alkyl groups in carboxylic acid led to a significant increase of lipophilicity (LogP = 0.82, 1.41, respectively), which plays a decisive role in the improvement of biological activities [9]. Hence, PCAEE has higher radical-scavenging activity by a better location at the lipid/aqueous phase interface where the oxidation occurs, and higher antimicrobial, antitumor, and anti-inflammatory effects [10,11]. Most of these actions take place in phospholipid bilayers or inside the cells, so it is essential to have information on the interaction of PCAEE with biological membranes. In this work, as a cell membrane model, we used multilamellar vesicles (MLV) and monolayers made of 1,2-dimyristoyl-sn-glycero-3-phosphocholine (DMPC) or 1,2-distearoyl-sn-glycero-3-phosphocholine (DSPC). We used differential scanning calorimetry (DSC) to study the interaction of PCAEE with MLV and Langmuir–Blodgett (LB) techniques to study the interaction of PCAEE with monolayers. MLV, when heated, exhibit a transition from the gel (Lβ) phase to the ripple phase, and then, a transition from the ripple phase to the liquid crystalline (Lα) phase [12,13,14,15]; these transitions can be revealed by DSC by measuring the associated thermodynamic parameters (transition temperature, T_m_, and enthalpy changes, ΔH). Compounds, interacting with the phospholipid bilayers, can modificate the lipid chain packing, that causes the variation of the transition thermodynamic parameters [16,17,18,19]. This behaviour can be analysed by the van’t Hoff model of the freezing-point depression of solutions [20,21]; Langmuir–Blodgett (LB) is commonly used to study the interaction between drugs and phospholipid monolayers, which represents a useful model for biomembranes. In monolayers, two thermodynamic variables, temperature and pressure, can be easily controlled [22,23,24]. An analysis of LB results may provide important information on the disposition and organization of compounds in lipid membranes. LB permits the obtaining of isotherms of phospholipids which, generally, are in the form of surface pressure/mean molecular area curves. The results can give indications on the ability of PCAEE to dissolve in the biomembrane models.

This study can provide new insights on the molecular interaction of PCAEE with components of cellular membranes. Parameters related to intermolecular interactions and their effects on properties of MLV and phospholipid monolayers can be easily obtained and related to the biological action of membrane-active drugs. 

## 2. Materials and Methods

### 2.1. Materials

Protocatechuic acid ethyl ester was purchased from Sigma-Aldrich Chimica S.r.l. (Milan, Italy). Synthetic DMPC and DSPC were obtained from Genzyme Pharmaceuticals (Liestal, Switzerland).

### 2.2. Multilamellar Vesicles Preparation

Stock solutions of DMPC, DSPC, and protocatechuic acid ethyl ester were prepared in chloroform/methanol (1:1, *v*:*v*). Aliquots of DMPC or DSPC solutions were distributed into glass tubes, such that there was the same amount of phospholipid in each tube; aliquots of a solution of protocatechuic acid ethyl ester were added to the tubes to obtain a defined molar fraction (0.00, 0.015, 0.03, 0.045, 0.09, and 0.12) of the compound with respect to phospholipid. The solvent was evaporated under nitrogen flow, and the resulting films were lyophilized to eliminate solvent residues. A quantity of 50 mM Tris solution (pH = 7.4) was added to the films to have 0.061 mmoles phospholipid/mL, and the samples were heated at 37 °C (DMPC) or 65 °C (DSPC) for 1 min, and successively shaken for 1 min; this procedure was repeated three times, and the samples were then kept at 37 °C or 65 °C for 1 h [25].

### 2.3. Encapsulation Efficiency

To determine the real amount of protocatechuic acid ethyl ether present in the phospholipid bilayers, samples of MLV were submitted to centrifugation at (60 × 10^3^) g for 1 h, using a Beckman L8-60M centrifuge at 4 °C. This temperature could affect the solubility of PCAEE. However, due to its lipophilic character, PCAEE incorporated in the MLV should remain inside them during the centrifugation. The amount of free protocatechuic acid ethyl ether present in the supernatant was detected by UV/Vis spectroscopy at 258 nm. The MLV encapsulation efficiency was evaluated by the following Equation (1):

(1) $$\mathrm{EE}\%\;=[({\mathrm{mgPCAEE}}_{\mathrm{tot}}-{\mathrm{mgPCAEE}}_{\mathrm{free}})÷{\mathrm{mgPCAEE}}_{\mathrm{tot}}]\times 100$$

### 2.4. DSC Analysis

A Mettler Toledo STARe system equipped with a DSC-822e calorimetric cell, and Mettler TA-STARe software was used. The sensitivity was automatically chosen as the maximum possible by the calorimetric system. The reference pan was filled with Tris solution. To calibrate the system in temperature and enthalpy changes, the procedure of the DSC 822 Mettler TA STARe instrument was followed.

### 2.5. Interaction between MLV and Protocatechuic Acid Ethyl Ester

A quantity of 120 µL of each MLV sample (corresponding to 0.007375 mmols of DMPC or DSPC) was transferred to a 160 µL aluminium pan, hermetically sealed and submitted, at least four times to check the results’ reproducibility, to the following DSC analysis: (1) a heating scan between 5 and 65 °C at 2 °C/min; (2) a cooling scan between 65 and 5 °C at 4 °C/min. Each experiment was carried out in triplicate to check the results’ reproducibility.

### 2.6. Surface Tension Measurements

A KSV minitrough apparatus including a 24,225 mm^2^ (available area) Teflon trough, two mechanically mobile coupled hydrophilic barriers (in Delrin), and a platinum surface-pressure sensor was used. A subphase consisting of 5mM Tris (pH 7.4) solution in ultrapure Millipore water (resistivity 18.2 MΩ cm) was used. Equimolar solutions (0.0012 mmol/mL) of DMPC and DSPC in chloroform and protocatechuic acid ethyl ester in chloroform/methanol (24.9:0.1; *v*:*v*) were prepared. Mixed DMPC/protocatechuic acid ethyl ester and DSPC/protocatechuic acid ethyl ester solutions were also prepared to obtain compounds with the following molar fractions with respect to the phospholipid: 0.015, 0.03, 0.045, 0.06, 0.09, 0.12, 0.25, 0.50, and 0.75. A quantity of 30 μL of the mixed solutions, as well as of the pure components, was spread drop by drop over the aqueous subphase by a Hamilton syringe. Before use, the Hamilton syringe was cleaned three times with chloroform and with the examined solutions. We then waited 15 min to permit the solvent to evaporate, and the films were compressed by the use of the mobile barriers at a rate of 10mm/min. Surface pressure as a function of molecular area isotherms was recorded. Before spreading the sample, to ascertain that no impurities were present on the subphase, blank experiments were run. The experiments were performed at a subphase temperature of 37 °C, which was kept constant by a thermostated circulating water bath [26]. Each run was repeated at least three times. 

## 3. Results and Discussion

**Figure 1:**
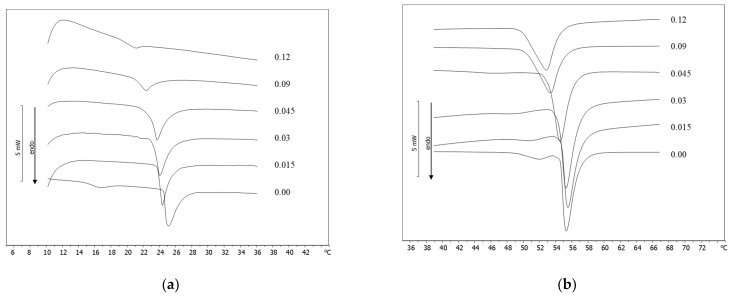
(**a**) Calorimetric curves, in heating mode, of DMPC MLV prepared with increasing molar fractions of PCAEE; (**b**) Calorimetric curves, in heating mode, of DSPC MLV prepared with increasing molar fractions of PCAEE.

Since Baghman’s study [27], vesicles have revolutionized pharmaceutical science, and several procedures have been developed to investigate the interactions of drugs and other bioactive molecules with biological membranes. For this reason, DSC has been developed as a useful tool to study these interactions and related phenomena. Phosphatidylcholine derivatives (DMPC and DSPC) were used to prepare a biological membrane model represented by MLV and to evaluate their interactions with PCAEE. MLV were prepared with and without increasing molar fractions of PCAEE. The calorimetric curves related to DMPC MLV are shown in Figure 1a. DMPC calorimetric curves show two peaks: a small peak associated with the transition from the gel phase to the ripple phase, called the “pre-transition peak”, and a main peak, associated with the transition from the ripple phase to liquid crystalline phase, called the “transition peak” [28]. Variations of the calorimetric curve (peak shape and/or transition temperature) of MLV prepared in the presence of the examined compound can indicate that the compound interacts with DMPC. Protocatechuic acid ethyl ester caused the disappearance of the DMPC MLV pre-transition peak at all the used molar fractions, indicating that the compound localized in the polar region of the lipid bilayers [29,30]; upon increasing the compound molar fraction, a gradual shift of the main peak towards lower temperatures and a concomitant broadening occurred. The presence of a unique peak suggested that protocatechuic acid ethyl ester was uniformly distributed in the DMPC bilayers. The calorimetric curve of DSPC MLV presented a main peak at 55.1 °C, associated with the transition from a gel to a liquid crystalline phase, a pretransition peak at about 51.8 °C, related to a change in tilt of the hydrocarbon chains, and a small shoulder next to the main peak. The presence of protocatechuic acid ethyl ester produced a marked variation on the calorimetric curve of DSPC MLV. At a low molar fraction of compound, the pre-transition peak decreased, and the main peak moved to a lower temperature. Starting from the 0.045 molar fraction, the pre-transition peak as well as the shoulder disappeared, and the main peak moved to a lower temperature and broadened (Figure 1b).

The amount of the percentage of PCAEE encapsulated in MLV is reported in Table 1. With regard to DMPC MLV, the amount of the percentage of PCAEE encapsulated in the MLV ranged from 85.72% to 88.79%, whereas DSPC MLV can encapsulate between 65.27% and 68.83% of the total amount of PCAEE. These results, although approximate, indicate that MLV are able to incorporate PCAEE, as observed from the DSC analysis, and that the amount of PCAEE does not affect the encapsulation efficiency.

**Figure 2:**
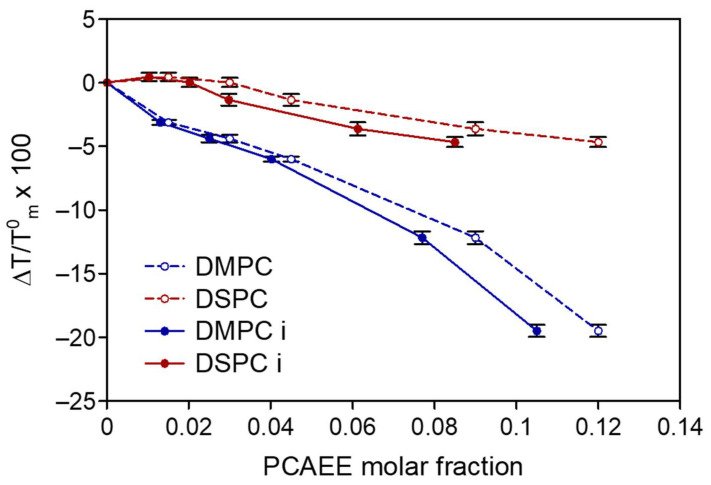
Transition temperature variation as ΔT/T^0^_m_ (ΔT = T_m_ − T^0^_m_; where T_m_ is the transition temperature of MLV with PCAEE, and T^0^_m_ is the transition temperature of MLV without PCAEE) of MLV as a function of the molar fraction of PCAEE. The dotted lines refer to the data obtained from the calorimetric curves. The continuous lines refer to the effect due to the real amount of PCAEE present in the MLV.

**Figure 3:**
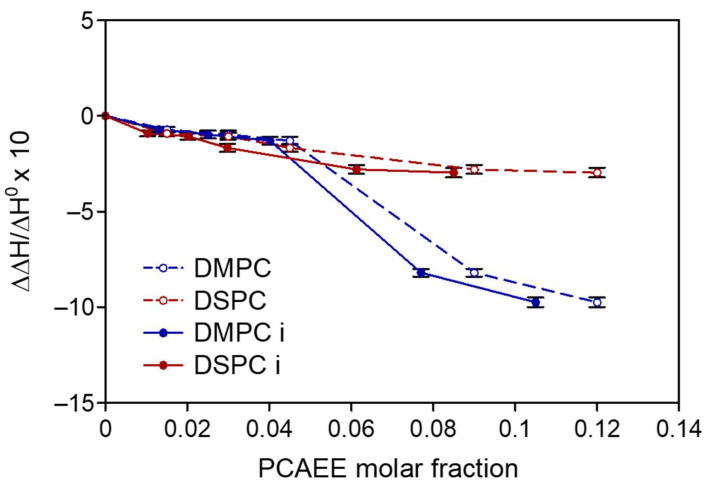
Enthalpy variation as ΔΔH/ΔH^0^ (ΔΔH = ΔH − ΔH^0^; where ΔH is the enthalpy variation of MLV with PCAEE, and ΔH^0^ is the enthalpy variation of MLV without PCAEE of MLV as a function of the molar fraction of PCAEE. The dotted lines refer to the data obtained from the calorimetric curves. The continuous lines refer to the effect due to the real amount of PCAEE present in the MLV.

The transition temperature changes obtained from the calorimetric curves are reported in Figure 2 (dotted lines) as ΔT/T^0^_m_ (ΔT = T_m_ − T^0^_m_, where T^0^_m_ is the temperature of the transition peak of MLV prepared without compound, and T_m_ is the temperature of the transition peak of MLV prepared in the presence of compound) as a function of the compound molar fraction present in the MLV bilayers. As stated, MLV were prepared with PCAEE at different molar fractions in the lipid aqueous dispersion; the compound could entirely localize in the lipid bilayers of the MLV, or could remain in part in the aqueous medium. Nevertheless, the observed effect has been attributed to the compound being present in the aqueous lipid dispersion and not really dissolved in the phospholipid membranes; then, it is worthwhile to determine the partition of PCAEE between the aqueous and the lipid phases to know the exact amount of compound present in the lipid phase of MLV. From the encapsulation, the exact amount of PCAEE in the lipid phase was found. The results were used to modify the data previously obtained (dotted lines) to determine the curves representing the effect of the real molar fraction of PCAEE present in the MLV (continuous lines of Figure 3). The tested compound caused the decrease of the phase transition temperature both of DMPC MLV and DSPC MLV, with a more pronounced effect on DMPC MLV. These results indicate that the compound caused a bilayer fluidization due to its insertion among the phospholipid molecules.

In Figure 3, the ΔH variations are reported as ΔΔH/ H^0^ (ΔΔH = ΔH − ΔH^0^, where ΔH^0^ is the enthalpy variation of MLV prepared without compound, and ΔH is the enthalpy variation of MLV prepared in the presence of each compound) as a function of the compound molar fraction present in the MLV samples. The dotted lines represent the effect of PCAEE present in the MLV aqueous dispersion, whereas the continuous lines represent the effect of PCAEE present in MLV bilayers. For both DMPC MLV and DSPC MLV, PCAEE causes a decrease of the ΔH. However, the decrease is more evident for DMPC MLV. If the compound is considered as an impurity dissolved in an ideal, two-dimensional solution, the lowering of the lipid bilayer transition temperature can be rationalized in terms of van’t Hoff’s depressing of freezing temperatures [22,31]. In this case, one should observe an almost linear decrease of the bilayer transition temperature with the concentration of the foreign molecule in the bilayer, while the associated enthalpy should remain constant. With regard to the temperature variation, this behaviour has been observed in Figure 2, which shows that the transition temperature decreases with the concentration of PCAEE. In Figure 3 where the enthalpy data (expressed as ΔΔH/H^0^) is presented, we observe a sharp decrease of the transition enthalpy only in the case of DMPC, while the enthalpy decrease is much smaller for DSPC. This behaviour indicates that the presence of PCAEE exerts a destabilization of lipid–lipid interactions. The strong interaction of PCAEE with the lipid chain prevents the highly cooperative melting of the lipid tails, causing the gel-to-fluid-phase transition of the bilayer to be less endothermic and less cooperative. As a result, the intensity of the calorimetric peak decreases, and the peak shape broadens. 

Therefore, we conclude that PCAEE is more soluble inside DMPC than inside DSPC, and it exerts a greater perturbation on the DMPC bilayer.

Lipid monolayers spread on air–water are simple models of membranes. They can be studied and manipulated in a Langmuir trough in which thermodynamic relationships between surface tension and surface area can be measured. Further information on the interaction between the compounds studied and the biomembrane models were obtained following the behaviour of compound/phospholipid mixed monolayers at the air/water interface, which was compared with that of the single-component monolayers. The molecular area/surface pressure isotherms were recorded at 37 °C (a temperature mimicking body temperature).

**Figure 4:**
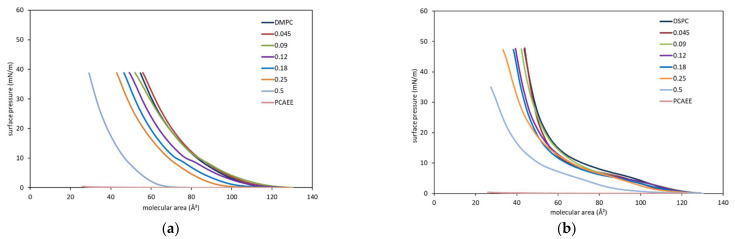
(**a**) Surface pressure/molecular area isotherms of DMPC, PCAEE, and DMPC/PCAEE mixed monolayers at the air–water interface at 37 °C; (**b**) Surface pressure/molecular area isotherms of DSPC, PCAEE and DSPC/PCAEE mixed monolayers at the air–water interface at 37 °C. The curves are the result of three experiments.

DMPC monolayers behave as a fluid membrane all along the compression isotherm curves. (See Figure 4a). In fact, a gas phase and a liquid expanded phase coexisted from 130 to about 118 Å^2^, and a liquid expanded phase is present for a molecular area lower than 118 Å^2^. PCAEE does not form monolayer, probably because it dissolves in the subphase. Low molar fractions (0.045–0.09) of PCAEE in the mixed monolayer do not cause significant variations in the isotherms, whereas high molar fractions cause the shift of the isotherms to lower mean molecular area values compared to the DMPC monolayer.

The DSPC isotherm shows a gas phase from 130 to 120 Å^2^, a liquid expanded phase from 120 to about 110 Å^2^, a liquid expanded-liquid compressed transition, and then, from about 60 Å^2^, a liquid condensed phase. The addition of PCAEE in the monolayer causes some variation in the isotherm. In particular, with the exception of 0.5 molar fraction of PCAEE up to about 15 mN/m, the isotherms are almost superimposable; for higher values of surface pressure, the higher the molar fraction of PCAEE, the bigger the shift to lower values of molecular area. At 0.5 molar fraction of PCAEE, the isotherm shifts to a lower molecular area (Figure 4b).

**Figure 5:**
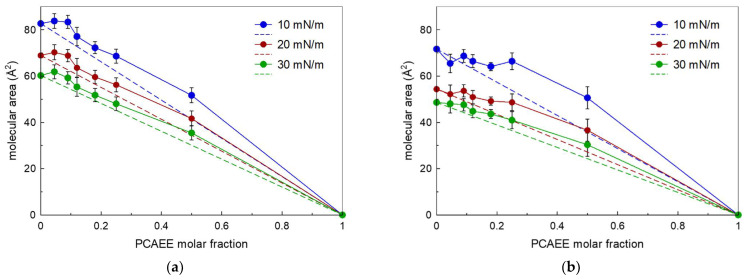
(**a**) Molecular area of the mixed monolayers of DMPC and PCAEE at the air–water interface plotted as a function of the mole fraction of compound at various values of surface pressures at 37 °C; (**b**) Molecular area of the mixed monolayers of DSPC and PCAEE at the air–water interface plotted as a function of the mole fraction of compound at various values of surface pressures at 37 °C.

Important information can be found by reporting, at different surface pressures, the mean molecular area as a function of the molar fraction of PCAEE present in the monolayer (Figure 5). The mean molecular area of a two-component monolayer is calculated by A = A_1_X_1_ + (1 − X_1_)A_2_, where A is the mean molecular area, X_1_ is the molar fraction of component 1, and A_1_ and A_2_ are the areas of the two pure components (phospholipids and PCAEE in this case) at the same surface pressure. Reporting in graph A as a function of X_1_, a straight line is obtained when the monolayer components are completely immiscible or possess an ideal miscibility [32,33]. Deviations from the straight line give indication on the interaction between the molecules. Positive deviation is an indication that repulsive interactions occur, whereas negative deviation indicates the attractive forces are established between the molecules. 

The relationship between the molecular area and molar fraction of PCAEE is not linear, indicating that PCAEE and the phospholipids (DMPC and DSPC) are not miscible, but rather showing a nonideal, mixed behaviour in the monolayer at the air–water interface.

Regarding DMPC, PCAEE caused at all the molar fractions a positive deviation that becomes less evident at 30 mN/m; this behaviour indicates the expansion of the monolayer and suggests that the PCAEE drug incorporates into the phospholipid monolayer. This behaviour could seem surprising because PCAEE is enabled to form a monolayer, and hence, it could dissolve in the subphase. However, other researchers [34] have found that monolayers containing both soluble and insoluble molecules may form despite the monolayer containing a soluble species (Figure 5a). 

In the PCAEE/DSPC mixed monolayers, mainly positive deviations occur, which becomes less evident as the surface pressure increases. Hence, PCAEE causes repulsive interactions among the molecules and an expansion of the monolayer (Figure 5b).

The reported data do not take into account the partition of PCAEE between lipids and water that could change by changing the molar fraction. So, the mean molecular area calculated in this way is an approximation. However, a consideration should be raised: When the mixed solutions are spread on the subphase to form the monolayers, as the subphase is static, the compound should remain anchored to the phospholipid molecules, unlike what would happen if the subphase were stirred. However, the aim of these experiments was first to evaluate if PCAEE affected the two phospholipids. The results indicate that PCAEE interacted with both of the phospholipids; in fact, if PCAEE had not interacted with the phospholipids, only a straight line should be obtained. Second, if the effect were different, the comparison of DMPC and DSPC behaviours highlights some differences: In PCAEE/DSPC mixed monolayers, the monolayer expansion is less evident than in PCAEE/DMPC mixed monolayers. An explanation could be given considering the length of the acyl chains of the phospholipid. The longer the acyl chains, the more the hydrophobic interactions which do not permit PCAEE to span the phospholipid molecules. PCAEE is a rather small molecule when compared with DMPC, and even more so when compared with DSPC. It could locate next the hydrophilic head and the proximal part of the hydrophobic tails spanning the phospholipid molecules but allowing the hydrophobic interaction among the tails which, being stronger in the DSPC, cause a less expansive effect. 

These results are in agreement with calorimetric data that show a lower effect of PCAEE on the DSPC MLV compared to the DMPC MLV. 

The obtained results highlight the usefulness of biomembrane models and the techniques used in this study in helping to understand the interaction of PCAEE in/with lipid assemblies, such as those of biological membranes. In addition, these findings may be of use to optimizing lipidic formulations of PCAEE.

## 4. Conclusions

These results show that PCAEE can be incorporated into simplified models of the cell membrane represented by MLV and monolayers made of DMPC and DSPC. In addition, the results suggest that the chain lengths of the phospholipids can play an important role in interactions with PCAEE. The information obtained is important because the interactions with the components of the cell membrane are essential for a compound to have a biological effect. Although further studies are required to obtain unambiguous conclusions, these results provide preliminary information on the bioavailability of PCAEE and could have a significant impact on understanding the interaction occurring between PCAEE and cell membranes during biological processes.

## Figures and Tables

**Table 1:** PCAEE encapsulation efficiency (EE) of DMPC MLV and DSPC MLV.

Sample	PCAEE Molar Fraction	EE%
DMPC MLV	0.0	-
	0.015	86.00 ± 5.00
	0.03	87.16 ± 6.21
	0.045	88.79 ± 3.81
	0.09	85.72 ± 7.12
	0.12	86.40 ± 2.80
DSPC MLV	0.0	-
	0.015	66.70 ± 3.20
	0.03	65.83 ± 4.51
	0.045	65.27 ± 4.32
	0.09	65.79 ± 2.82
	0.12	68.36 ± 3.91

## Data Availability

Data were generated at the Department of Drug and Health Science, University of Catania. Data supporting the results of this study are available from the corresponding authors on request.
